# Understanding the sociodemographic factors associated with intention to receive SMS messages for health information in a rural area of Bangladesh

**DOI:** 10.1186/s12889-021-12418-9

**Published:** 2021-12-30

**Authors:** Fakir M Amirul Islam, Elisabeth A Lambert, Sheikh Mohammed Shariful Islam, M Arzan Hosen, Bruce R Thompson, Gavin W Lambert

**Affiliations:** 1 School of Health Sciences, Swinburne University of Technology, John Street, Hawthorn, VIC 3122 Australia; 2 Organization for Rural Community Development (ORCD), Dariapur, Narail 7500 Bangladesh; 3 Iverson Health Innovation Research Institute, Swinburne University of Technology, John Street, Hawthorn, VIC 3122 Australia; 4 Institute for Physical Activity and Nutrition (IPAN), Faculty of Health Deakin University, Burwood, VIC 3125 Australia

**Keywords:** Mobile phone, Ownership, Reading SMS, Rural area, Bangladesh

## Abstract

**Background:**

The use of digital interventions for managing chronic diseases is significantly increasing. The aim of this study was to estimate the proportion of ownership of a mobile phone, and factors associated with the ability to read and access SMS delivered health information, and willingness to pay for it among people with hypertension in a rural area in Bangladesh.

**Methods:**

Data were collected from 307 participants aged 30 to 75 years with hypertension from a rural area in Bangladesh from December 2020 to January 2021. Outcome measures included ownership of a mobile phone, ability to read SMS, willingness to receive and pay for health information by SMS. Associated factors included age, gender, level of education, occupation, and socioeconomic status. We used regression analysis to identify variables associated with the outcome variables.

**Results:**

Overall, 189 (61.6%) people owned a mobile phone which was higher in men (73.3% vs. 50%, *p* < 0.001), younger people (82.6% aged 30–39 years vs. 53.5% aged 60–75 years, *p* < 0.001). Of the total participants, 207 (67.4%) were willing to receive SMS, and 155 (50.5%) were willing to pay for receiving SMS for health information. The prevalence was significantly higher among professionals (odds ratio (OR), 95% confidence interval (CI): 4.58, 1.73–12.1) and businesspersons (OR 3.68, 95% CI 1.49–9.10) compared to farmers, respectively. The median (interquartile range [IQR]) of willingness to pay for health information SMS was 10 (28) Bangladesh Taka (BDT) (1 BDT ~ 0.013 US$), and there were no specific factors that were associated with the willingness of any higher amounts of payment. In terms of reading SMS of people who own a mobile, less than half could read SMS. The proportion of people who could read SMS was significantly higher among men, younger people, educated people, middle class or rich people, professionals or businesspersons. Of people who could read SMS, the majority read SMS occasionally.

**Conclusion:**

A significant proportion of people are unable to read SMS. However, people are willing to receive and pay to receive SMS for health information. Education and awareness programs should be conducted among targeted groups, including people with low education and women.

## Background

Hypertension is a major risk factor of cardiovascular diseases and a leading cause of death globally, accounting for 10.4 million deaths per year, of which 85% occur in low-middle income countries [1, 2]. Managing hypertension appropriately requires a long-term care plan, including advice and support for adherence to a healthy lifestyle and treatment for better health outcomes. Unfortunately, non-adherence to disease management plans remains a serious challenge globally, with the situation worse in developing countries [3–9].

Mobile health (mHealth) technologies have been playing a key role in managing disease outcomes including in conditions such as diabetes, hypertension and other cardiovascular diseases, maternal health and psychological disorders [10–14]. The use of mHealth empowers patients, health workers and health system managers to manage care efficiently and effectively through different ways, including providing online guidelines and referral services, reminders and self-management and medication adherence. While studies indicate that mHealth systems can increase patients’ satisfaction with quality health care [12, 15–20], other reports have provided mixed evidence regarding the health benefits of mHealth [21].

The availability of mHealth initiatives is significantly higher in high-income countries [22] but the use of mHealth is growing in low-income countries including in underserved rural areas [17, 19, 22–27]. It is also expected that mHealth will continue to grow due to the increasing availability and accessibility of smartphones [24, 28]. Of mHealth platforms, the most used tool is the short message system (SMS), predominantly focused on patient-centred outcomes [27]. Currently, 67% of the world population, and around 50% in the Asia Pacific region, have a mobile or smartphone, an increase of 40% from 2016 to 2020 [29].

Bangladesh is confronting a significant increase in chronic diseases including hypertension [30, 31] with the pooled prevalence of hypertension was reported to be 41% with a cut-off value of ≥ 130/80 mmHg and/or use of antihypertensive medications [31]. Nineteen percent of the population use a smartphone and, despite the problems in the usability of mhealth information due largely to network access in rural and remote areas [25, 29, 32], the country has made significant progress in the use of mHealth [19, 33–35]. In recent years, several studies have been conducted to study the use of mHealth in Rural Bangladesh [34–36]. Factors associated with the use of mHealth reported that young people (age < 30 years), irrespective of their socio-demographic variation, have greater access to mobile phones, and better knowledge and greater intention to use mHealth services for managing their health [36]. Other essential factors include gender, level of education and socioeconomic status, which influence ownership of mobile phones and hence may impact on the delivery and uptake of mHealth initiatives. Previous studies reported that the proportion of mobile phone use was almost double among men compared to women in rural Bangladesh, with the difference occurring irrespective of their socio-economic status. While men were more aware of available mHealth services there occurred no difference between gender in intention to use services [34]. Given that problems related to network access are still significantly higher in rural and remote areas compared to urban areas [25, 29, 32], and that less than 50% of the rural population in Bangladesh own a mobile phone [34, 36], mHealth solutions are yet to be developed and implemented on a mass scale. The objectives of this study were to estimate the proportion of, participants with high blood pressure (i) who own mobile phones, (ii) who are willing to receive SMS, (iii) who are willing to pay for SMS to receive health information, (iv) who can read, and who read SMS, and (v) how much are individuals willing to spend to receive health information. Sociodemographic factors associated with these objectives were also examined.

## Methods

### Study design and population

We recruited 307 participants aged between 30 to 75 years to a cluster randomised control trial to lower blood pressure by attention to lifestyle [37]. Data were collected from the Banshgram Union of the Narail District in Bangladesh, located approximately 200 km southwest of Bangladesh's capital city. Participants from the cross-sectional Bangladesh Population-based Diabetes and Eye Study [38, 39] previously diagnosed with stage 1 hypertension [40] were the source for the current investigation. To understand the study location, Bangladesh is a country of over 163 million people divided into 64 districts. Each district is divided into Upazilas (sub-districts), and each Upazila is divided into Unions which consist of 15–20 villages [41].

### Sample size calculation

A previous study reported that 45% of people in a rural district in Bangladesh owned a mobile phone [36]. To estimate the proportion of people who owned a mobile phone the sample size of 307 was adequate with a statistical power of at least 90% and type I error 0.05 with 7.5% points above or below the estimated proportion. If we can estimate 50% of people owned a mobile phone, i.e., 150 people had a mobile phone, the sample size of 150 was adequate with a statistical power of at least 90% and type I error 0.05 with 10% points above or below the estimated proportion of people who can read SMS.

### Recruitment and data collection

The Organization for Rural Community Development, a local non-government organization in the Narail district of Bangladesh, facilitated recruitment. The Organization for Rural Community Development investigators and trained data collectors communicated with the potential participants over the telephone or by direct contact. Upon establishment of contact with potential participants, they were assessed for inclusion and exclusion. The inclusion criteria were: (i) clinic blood pressure more than or equal to 130/80 mm Hg who were not taking medication, (ii) blood pressure < 130/80 but using anti-hypertensive medication for a minimum of six weeks, and (iii) living in the Banshgram Union only. The exclusion criteria were: (i) aged > 75 years of age, (ii) pregnant, (iii) advanced CVDs or had any serious condition that restricted their participation in the study

Data were collected from face-to-face interviews with the Organization for Rural Community Development investigators and four trained data collectors who took part in data collection. An equal proportion of men and women subjects were recruited. The chief investigator conducted four zoom meetings to train the data collectors and local investigators on the steps to be followed to conduct this research. We conducted a pilot study before the main data collection to familiarize the data collectors with all study procedures. The data collection process was monitored by the Organization for Rural Community Development investigator for quality assurance. The Organization for Rural Community Development investigator revisited 10% of the household to cross-check data quality. We collected the baseline data for this study from December 2020 to January 2021. After baseline data collection from all participants, the study location-the Banshgram Union comprised of 18 villages [41] was divided into two clusters, cluster 1 with nine villages with 156 participants- the intervention arm, and cluster 2 with the remaining nine villages, which had 151 participants-the control arm. The intervention arm involves (i) delivering a blended learning education program, including delivering printed materials, and (ii) weekly phone calls to the team leaders of each of ten teams comprised of 12–16 participants each. The intervention is an educational program focused on awareness of a healthy lifestyle, including motivation to participate in regular physical activity, community engagement and frequent contact with health professionals.

The study location, the sources population's demographic characteristics, and the cluster RCT have been described in detail [37, 38].

## Outcome Variables:

### The outcome measures for this study were:

1. Mobile phone ownership with two possible responses, “yes” and “no”.
2. Willingness to receive SMS for health information with two possible responses, “yes” and “no”. This question is to measure peoples’ motivation to receive SMS for health information.
3. Willingness to pay to receive SMS for health information with two possible responses, “yes” and “no”.
4. Can read SMS with two possible responses, “yes” and “no”. This question did not differentiate the cause, which could include issues around literacy, eye sight or any other reasons that could be related to reading SMS.
5. If they can read SMS, how do they read SMS (practice of reading SMS) with possible responses, “read all”, “if they consider the SMS is important”, “SMS from known people”, “read SMS occasionally” and “never read”.
6. If they are willing to pay, intended amount of payment to receive SMS for health information (the amount in Taka, Bangladeshi currency).

### Exposure variables

Exposure variables for this study were age, categorized into 30–39, 40 to 59 years and 60-75 years. The age category 30–39 years was considered to be younger adults, participants aged 40–59 years were adults, and people of age 60–75 years were considered to be older adults. We postulated that there could have been differences between the younger adults (age 30–39 years) and older adults (60-75 years). The level of education was categorized as no schooling, primary to high school (grade 1 to 9), secondary school certificate or any higher-level education. Socio-economic status was assessed according to Cheng et al.[42]. Since most participants had no taxable income, a crude measure of SES was used following Cheng et al. [42] where we asked whether "over the last twelve months, in terms of household food consumption, how would you classify your socio-economic status?" The possible answers were: (i) Insufficient funds for the whole year, (ii) Insufficient funds some of the time, (iii) Neither deficit nor surplus (balance) and (iv) Sufficient funds most of the time. We re-categorised these four categories as poor who had insufficient funds at least some of the time-categories 1 and 2, and middle class or rich who had neither deficit nor surplus (balance) or better SES- categories 3 and 4. Occupations were categorised as: farmer, housewife, businessperson, labourers which included digging soils, pulling rickshaw or any laborious works, and professionals including government and non-government employees.

### Validation of the instrument

We prepared the instrument first in English. It was then translated into Bengali separately by a local senior educator and the principal investigator. Their versions were then combined based on discussion and agreement. The questionnaire was then pilot-tested with ten eligible adults who were not included in the final study. We performed the pilot test to assess the comprehension, wording, and appropriateness of the measures. While the measures have not been formerly validated in Bangladesh, previous studies used similar tools to collect data on mobile ownership, and ability to read and willingness to pay for SMS in individuals with diabetes [36, 43].

### Statistical analysis

Socio-demographic characteristics including gender, age, level of education, socio-economic status and occupation were reported using frequency tables. We performed bivariate associations to test the hypotheses that none of the factors, including age group, gender and level of education were associated with the outcome variables. The outcome variables were: (1) mobile phone ownership, (2) willing to receive SMS for health information, (3) willing to pay to get SMS for health information, (4) can read SMS, (5) practice of reading SMS, and (6) antended amount of payment. We used the binary logistic regression technique to estimate the odds ratio (OR) and 95% confidence intervals (CI) for the outcome variables. Intention to spend money to receive health information was presented as a proportion of people willing to pay. A bivariate test of associations was performed to test the hypotheses that none of the factors were associated with willingness to pay for receiving health information. The amount of intended money spent was presented as median, quartile-one and quartile-four, and minimum and maximum amount using descriptive statistics. Since the amount of intended money spent was skewed, the non-parametric Wilcoxon sign rank test was performed to assess the associations of sociodemographic factors with intended money spent. Statistical software SPSS (SPSS Inc, version 27) was used for the analysis.

## Results

### Study participants

Table 1 shows the socio-demographic characteristics of the study participants. The study participants comprised an almost equal proportion of men and women. Around half of the participants were 40–59 years of age, 15% were 30–39 years of age. One-fifth of the participants had completed a secondary school certificate or above, whereas one-third had no schooling. Half of the participants were homemakers, about one-fifth were farmers, and 18% were professionals (government or private).

**Table 1:** Socio-demographic characteristics of the study participants

	Number	Percentage
Gender		
Woman	154	50.2
Man	153	49.8
Age group, years		
30–39	46	15.0
40–59	160	52.1
60–75	101	32.9
Level of Education		
No education	99	32.4
Primary to high school	148	48.4
Secondary School Certificate or above	59	19.3
Socioeconomic status		
Poor or very poor	92	30.1
Middle class	214	69.9
Occupation		
Homemaker	146	8.3
Businessperson	24	8.3
Labourer	7	2.4
Farmer (Agriculture)	59	20.4
Professional (government. or private)	53	18.3

Table 2 shows the proportion participants who owned a mobile phone and the factors associated with mobile phone ownership, willingness to receive SMS and willing to pay for receiving health information by SMS. In the total sample, 189 (61.6%) owned mobile phones, and all received SMS. There was a significant difference in mobile ownership between gender (men: 73.2% vs. women: 50%, *p* < 0.001), age group (30–39 years of age: 82.6%, 40–59 years: 60.6% and 60–75 years: 53.5%, *p* = 0.003 for trend), level of education (no education: 40%, primary to below secondary school certificate (SSC): 64.2%, SSC or above: 91.5%, *p* < 0.001 for trend), SES (poor: 46.7% vs. middle class or rich: 67.8%, *p* = 0.001) and occupation (Farmer: 68.2%, housewife: 52.1%, professionals including government and non-government employees: 64.2% and businessperson: 87.5%, *p* = 0.003). After adjustment for age, gender, level of education, socio-economic status and occupation, logistic regression analyses revealed that men compared to women, odds ratio (OR) (95% confidence interval (CI): 2.60 (1.46, 4.62), younger age compared to older age, for example, age 30–39 years compared to 60–75 years: OR 3.80 95% CI (1.40, 10.32) were associated with a higher prevalence of mobile phone ownership. Of the total participants, 67.4% were willing to receive SMS for health information, with professionals (88.7%) more willing to receive SMS. Of the total participants, 50.5% were willing to pay for receiving health information by SMS. The proportion of willing to pay for receiving health information was higher among professionals (71.7%).

**Table 2:** Mobile ownership, willingness to receive and pay for health information by SMS and their associated socio-demographic factors

		Mobile ownership		Willing to receive SMS		Willing to pay	
		n (%)	OR (95% CI)^a^	n (%)	OR (95% CI)^a^	n (%)	OR (95% CI)^a^
Total	307	189 (61.6)		207 (67.4)		155 (50.5)	
Gender							
Woman	154	77 (50)	1.0 (ref)	94 (61.0)	1.0 (ref)	72 (46.8)	1.0 (ref)
Man	153	112 (73.2)	2.60 (1.46, 4.62)	113 (73.9)	1.67 (0.96, 2.93)	83 (54.2)	1.35 (0.82, 2.25)
* P*		< 0.001		0.02		0.19	
Age group, years							
30–39	46	38 (82.6)	3.80 (1.4, 10.32)	36 (78.3)	1.85 (0.75, 4.59)	25 (54.3)	1.36 (0.63, 2.95)
40–59	160	97 (60.6)	1.89 (1.03, 3.47)	110 (68.8)	1.69 (0.94, 3.02)	83 (51.9)	1.36 (0.80, 2.33)
60–75	101	54 (53.5)	1.0 (ref)	61 (60.4)	1.0 (ref)	47 (46.5)	1.0 (ref)
* P*		0.003		0.09		0.60	
Level of education							
No education	99	40 (40)	1.0 (ref)	52 (52.0)	0.53 (0.3, 0.92)	42 (42.4)	1.0 (ref)
Primary to below SSC	148	95 (64.2)	2.1 (1.20, 3.68)	105 (70.9)	1.7 (0.73, 3.95)	78 (52.7)	1.35 (0.79, 2.31)
SSC or above	59	54 (91.5)	7.98 (2.78, 22.9)	50 (84.7)	1.0 (ref)	34 (57.6)	1.47 (0.7, 3.11)
* P*		< 0.001		< 0.001		0.13	
SES							
Poor	92	43 (46.7)	1.0	51 (55.4)	1.0 (ref)	40 (43.5)	1.0
Middle class or above	214	145 (67.8)	1.85 (1.06, 3.2)	155 (72.4)	1.74 (1.02, 2.97)	115 (53.7)	1.38 (0.83, 2.29)
* P*		0.001		0.004		0.10	
Occupation							
Farmer	66	45 (68.2)	1.0 (ref)	43 (65.2)	1.0 (ref)	29 (43.9)	1.0 (ref)
Housewife	146	76 (52.1)	0.51 (0.27, 0.93)	87 (59.6)	0.44 (0.04, 5.08)	66 (45.2)	0.75 (0.11, 5.06)
Professional	53	34 (64.2)	0.84 (0.39, 1.79)	47 (88.7)	3.95 (1.25, 12.5)	38 (71.7)	3.68 (1.49, 9.10)
Businessperson	24	21 (87.5)	3.27 (0.88, 12.18)	20 (83.3)	1.98 (0.57, 6.88)	14 (58.3)	2.05 (0.74, 5.66)
* P*		0.003		< 0.001		0.004	

^a^Odds Ratio (95% confidence interval) adjusted for the variables (gender, age, education levels, SES and occupation) in the model

Table 3 shows the proportion of people who could read SMS and the associated factors and the practice of reading SMS. Of 189 people who owned a mobile device and received SMS, only 45% noted that they could read SMS, which was significantly higher in men, younger people, educated people, and those middle class or rich, professionals or businesspersons. Of those who could read SMS (*n* = 85), one-fifth would read all the SMS, 32.9% would read if they considered it was important, 31.8% would read occasionally, and 8.2% would never read. Younger people of age 30–39 years (35.5%) were more likely to read all of the SMS.

**Table 3:** Factors associated with ability to read and practice of reading SMS, and their associated socio-demographic factors

		Can read SMS who own a mobile			Practice of reading SMS†				
	Ownership of a mobile	n (%)	OR (95% CI)^a^	Can read	Read all	If it is important	From known people	Occasionally	Never read
Total	189	85 (45.0)		85	19 (22.4)	28 (32.9)	4 (4.7)	27 (31.8)	7 (8.2)
Gender									
Woman	77	23 (29.9)	1.0 (ref)	23	4 (17.4)	5 (21.7)	0	9 (39.1)	5 (21.7)
Man	112	62 (55.4)	3.84 (1.5, 9.78)		15 (24.2)	23 (37.1)	4 (6.5)	18 (29.0)	2 (3.2)
* P*		0.001			< 0.001				
Age group, years									
30–39	38	31 (81.6)	17.3 (3.99, 75.3)	31	11 (35.5)	13 (41.9)	0	5 (16.1)	2 (6.5)
40–59	97	36 (37.1)	2.06 (0.77, 5.53)	36	4 (11.1)	10 (27.8)	2 (5.6)	17 (47.2)	3 (8.3)
60–75	54	18 (33.3)	1.0 (ref)	18	4 (22.2)	5 (27.8)	2 (11.1)	5 (27.8)	2 (11.1)
* P*		< 0.001			< 0.001				
Level of education									
No education	39	0	–						
Primary to below SSC	95	38 (40.0)	1.0 (ref)	39	4 (10.3)	12 (30.8)	2 (5.1)	16 (41.0)	5 (12.8)
SSC or above	54	46 (85.2)	5.52 (2.11, 14.4)	46	15 (32.6)	16 (34.8)	2 (4.3)	11 (23.9)	2 (4.3)
* P*		< 0.001			0.004				
SES									
Poor	43	9 (20.9)	1.0	9	2 (22.2)	3 (33.3)	0	2 (22.2)	2 (22.2)
Middle class or above	145	74 (51)	3.74 (1.34, 10.4)	75	16 (21.3)	25 (33.3)	4 (5.3)	25 (33.3)	5 (6.7)
* P*		< 0.001			0.54				
Occupation									
Farmer	48	16 (35.6)	1.0 (ref)	17	2 (11.8)	5 (29.4)	2 (11.8)	7 (41.2)	1 (5.9)
Housewife	76	23 (30.3)	0.72 (0.33, 1.55)	23	4 (17.4)	5 (21.7)	0	9 (39.1)	5 (21.7)
Professional	34	25 (73.5)	4.58 (1.73, 12.1)	25	7 (28.0)	9 (36.8)	2 (8.0)	7 (28.0)	0
Businessperson	21	13 (61.9)	2.68 (0.92, 7.78)	13	3 (23.1)	6 (46.2)	0	3 (23.1)	1 (7.7)
* P*		< 0.001			0.26				

^a^Odds Ratio (95% confidence interval) adjusted for the variables (gender, age, education levels, SES and occupation) in the model; †Fisher’s exact test was used for any cell frequencies less than five

Table 4 shows the amount of Bangladeshi Taka that people were willing to pay to receive health information by SMS. People were willing to pay Bangladeshi Taka (BDT) 10 (1 BDT ~ 0.013 US$) with the interquartile range 25–53, minimum 5 (0.065 US$) and a maximum of 500 Taka (6.5 US$) per month. None of the demographic factors were significantly associated with higher or lower payment intention.

**Table 4:** Intended amounts to pay to receive health information by SMS and the associated sociodemographic factors

Characteristics	Willing to pay, N (%)	Median	Q1	Q3	Min	Max	*P*^a^
Total	155 (50.5)	10	25	53	5	500	
Gender							0.34
Woman	72 (46.5)	10	5	20	5	300	
Man	83 (53.5)	10	5	20	5	500	
Age group, years							0.74
30–39	25 (16.1)	10	5	20	5	200	
40 – 59	83 (53.5)	10	5	20	5	500	
60–75	47 (30.3)	5	5	20	5	500	
Level of Education							0.06
No education	42 (27.3)	5	5	10	5	300	
Primary to high school	78 (50.6)	10	5	20	5	300	
SSC or above	34 (22.1)	10	5	30	5	500	
Socio-economic status							0.87
Poor	40 (25.8)	10	5	20	5	300	
Middle class or higher	115 (74.2)	10	5	20	5	500	
Occupation							0.46
Farmer	29 (19.7)	10	5	10	5	500	
Housewife	66 (44.9)	10	5	20	5	300	
Professional	38 (25.9)	10	5	20	5	300	
Businessperson	14 (9.5)	8	5	10	5	500	

^a^*p* for non-parametric Wilcoxon sign rank test

## Discussion

Ownership of mobile phones and the ability to read and understand SMSs or receive voice messages are the key elements for successfully implementing mHealth technology [44]. The significant findings from this study include: (1) that in rural Bangladesh, there is a gap in ownership of mobile phones, with about half of the women and half of the older people owning a mobile, (2) irrespective of socio-demographic factors, the willingness to receive SMS for health information was high, (3) half of the participants were willing to pay to receive health information, (4) individuals were willing to pay between 5 to 500 Taka, with the majority were willing to pay 10 Taka, (5) less than half of the people who use their mobile can read SMS, and this percentage is less than one-third among women, housewives, farmer and people from low SES, and (6) only a small percentage of people read all the SMSs, with lower numbers seen in women, farmers, housewife and older people.

In our study, men, younger people, people with higher education or people with higher SES were more likely to own mobile phones compared to women, older people or people with no education, which are consistent with the previous studies in Bangladesh [34, 36, 45] and other low-middle income countries [15, 46]. The use of mHealth, including sending text messages, is effective in managing hypertension through varieties of ways, including by providing educational information on a healthy lifestyle, self-monitoring of blood pressure and through providing a reminder of medication adherence. The use of mHealth has been reported to effectively control blood pressure in low-middle income countries, including China and Brazil [47–49]. Our finding that less than half of our population could read SMS identifies further barriers in implementing mHealth programs. Extrapolating to the broader rural population, from our data, if 50% of women own a mobile phone but only 30% can read SMS, only 15% of adult women can be considered to have full access to the potential mHealth benefits enabled by mobile technology.

In low-middle income countries, mobile phone ownership has increased significantly since 2014. In South Asia, women are 28% less likely to own a mobile phone than men, and are 58% less likely to use mobile internet [50]. In this study, we found that women are 23% less likely to own a mobile phone than men, which is consistent with previous findings in South Asia [50]. In previous studies among patients with diabetes conducted in Nigeria and Bangladesh, comprising of approximately 60% of women, it was reported that almost everyone owned a mobile phone [43, 51]. The higher proportion of mobile ownership in previous studies may be due to the financial or educational variation to access mobile and internet facilities, as both studies were urban based. In the current study which was conducted in a rural area, a low level of education, being a housewife and possibly without having a regular income can be reasons for less ownership of mobile devices and impact on one’s ability to read SMS. The differences observed in the present report compared with previous studies may be due to differences in education levels. In the current study, 19% of participants had a secondary school certificate or above, compared to 49% with tertiary education in the Nigerian study [51] and 71% with secondary or above education level in an urban based tertiary hospital in Bangladesh [43]. Consistent with previous studies [34, 46], we found no gap between mobile phones ownership amongst men and women who have at least a secondary school certificate. Although women and girls continue to face a wide range of discriminations, Bangladesh has made significant progress on poverty alleviation, gender equality and women’s empowerment over the past two decades [52, 53]. A study conducted by Khatun et al. [34] in 2012–2013 in a rural area reported that 61.8% of men and 34.4% of women had a mobile phone compared to 73% of men and 50% of women in our study. This indicates a positive trend in mobile phone ownership over time, and the gap in ownership of mobile phone between men and women is narrowing, consistent with previous findings [50].

In terms of reading SMS, a study conducted in Nigeria among participants with diabetes reported that three-quarters of participants were able to read SMS, with no difference between gender [51]. However, less than three-quarter of men and one-quarter of women in our study could read SMS, which is similar to that reported in a previous study in Bangladesh among patients with diabetes [43]. In terms of willingness to receive SMS for health information, our results are similar to those reported in previous studies [43, 51].

Another challenge in delivering effective mHealth solutions is related to the attitude of individuals towards reading SMS. In this study, only a small proportion of participants read the full content of SMSs. In contrast with the findings that there was no difference in ownership of mobile phone between men and women among those with higher education, the proportion of women who read SMS was lower than that in men among the same education group, which is consistent with a previous study [43]. Empirical evidence suggests that there are significant gender differences in exposure to technology where women's participation is lower than that of men [54]. In our study, a disadvantaged position among women is evident reflecting the findings that 57% of women compared to 44% of men were reported to be poor, 15% of women compared to 85% of men were professionals and 61% of women compared to 39% of men had no education. This indicates that the intersectionality of confounding factors conspire to disadvantage women and influence their ability to own mobile phones and read SMS.

In our study and consistent with others, many participants were willing to receive and pay for mobile-based health services, [43, 51] indicating a positive attitude towards SMS in considering its benefits for managing health. Since the participants already were diagnosed with hypertension, they are likely to be aware of medication use or complications associated with the disease. They are willing to receive health information to manage their health and well-being.

Our findings show that younger people are more likely to have mobile phones, read SMSs and are willing to pay for receiving health information. Importantly, engaging the younger generation earlier holds promise for developing and implementing mHealth initiatives in the future [36]. To increase involvement in mHealth, ownership of a mobile device is not the only barrier. Level of education, health literacy and access to services provide additional inertia. It is important to co-design mHealth interventions appropriate for the individual end-user, considering their demographic details, including age, gender, education, and access to services [55]. While the use of the internet may be an option to provide health information and personalized service, access, especially in rural areas, is not guaranteed. For example, in the UK, a study by Dobson et al. [56] reported that more than 90% of their participants used the internet to their mobiles compared to 33.5% who used the same mode of access in Nigeria [51]. In Bangladesh, 54.8% of urban and 34.8% of rural people use the internet on their mobile phone [57]. Mobile phone-based health services are reported to be further restricted by intermittent electricity to keep charge on mobile phones, poor network connections and insufficient financial power to regularly buy credits, especially in low-middle income countries [43, 57].

In Bangladesh, a major barrier in the health system has been identified as the shortage of qualified doctors, especially in rural areas [58, 59]. The use of mHealth can potentially address this shortage [34, 36]. Continued development in the rural area is needed to facilitate greater ownership of mobile phones and education to read SMS for the benefits of mHealth to be achieved in women from socio-disadvantaged regions [34, 36]. Our findings are significant in that they demonstrate that ownership of a mobile phone is not the sole barrier to access mHealth services. Instead, the ability to read or attitudes and habits of reading SMS messaging are important. Although we had not included the option of choosing either SMS or voicemail or phone calls, a previous study showed that participants preferred phone calls and text messages when receiving information associated with education [51]. Participants with no education or a primary education level had the preference for receiving phone calls [51]. Given the low education level among older adults and women in our study, direct phone calls may be a more helpful option than sending SMS. The preference of phone calls, although more labour intensive, could assure confidentiality as well as direct contact with providers rather than relying on SMS. Thus, our study provides further insight into the barriers to the use of mHealth in rural areas of Bangladesh, and a possible solution of sending voice mail could be an option but needs further study.

Our study has several strengths: firstly, data was collected face-to-face whilst maintaining social distancing and other health and safety issues. The study had an almost equal number of men and women. However, the study has several limitations. Firstly, the results are from the baseline data recruited for a cluster RCT, and thus the sample size is small. Secondly, the study has been conducted in one district, limiting the generalisation at the national level. However, the rural population in terms of socio-demographic and education level is very similar across Bangladesh [60].

## Conclusions

In rural Bangladesh mobile phone ownership is not high. The current situation makes it difficult to adopt mHealth strategies for managing chronic diseases. Although less than one-third of women, older people and people from low SES can read SMS, a small proportion of people read all the SMS. However, a high proportion of people are willing to receive SMS for health information and willing to pay for it. To optimise the use of mHealth, appropriate techniques including sending SMS or phone calls should be arranged, and education and awareness programs should be conducted among targeted groups, including people with low education and women.

## Data Availability

The corresponding author will make the data used for this study available on reasonable request. Authors declare no conflict of interest.

## Abbreviations

ORCD: Organization for Rural Community Development
SMS: Short message system
SES: Socioeconomic status
CI: Confidence Interval
OR: Odds Ratio
